# Self-organized insulin-producing β-cells differentiated from human omentum-derived stem cells and their in vivo therapeutic potential

**DOI:** 10.1186/s40824-023-00419-1

**Published:** 2023-08-29

**Authors:** Ji Hoon Jeong, Ki Nam Park, Joo Hyun Kim, KyungMu Noh, Sung Sik Hur, Yunhye Kim, Moonju Hong, Jun Chul Chung, Jae Hong Park, Jongsoon Lee, Young-Ik Son, Ju Hun Lee, Sang-Heon Kim, Yongsung Hwang

**Affiliations:** 1 Soonchunhyang Institute of Medi-Bio Science (SIMS), Soonchunhyang University, Cheonan, Chungnam-Do 31151 Republic of Korea; 2 Department of Integrated Biomedical Science, Soonchunhyang University, Asan, Chungnam-Do 31538 Republic of Korea; 3 Department of Otorhinolaryngology-Head and Neck Surgery, Soonchunhyang University Bucheon Hospital, Soonchunhyang University College of Medicine, Bucheon, 14584 Republic of Korea; 4 Department of Otorhinolaryngology-Head and Neck Surgery, Soonchunhyang University Cheonan Hospital, Cheonan, 31151 Republic of Korea; 5 Department of Surgery, Soonchunhyang University Bucheon Hospital, Bucheon, 14584 Republic of Korea; 6 Department of Otorhinolaryngology-Head and Neck Surgery, Samsung Medical Center, Sungkyunkwan University School of Medicine, Seoul, 06351 Republic of Korea; 7 Department of Bionano Engineering, Center for Bionano Intelligence Education and Research, Hanyang University, Ansan, 15588 Republic of Korea; 8 Center for Biomaterials, Biomedical Research Institute, Korea Institute of Science and Technology, Seoul, 02792 Republic of Korea; 9 Department of Bio-Med Engineering, KIST School, Korea University of Science and Technology, Seoul, 02792 Republic of Korea

**Keywords:** Pancreatic β-cells, Cell adhesion, Insulin-producing cells, Cell-to-cell interaction, Fibroblast growth factor 2, Stem cell differentiation, Streptozotocin-induced diabetic models

## Abstract

**Background:**

Human omentum-derived mesenchymal stem cells (hO-MSCs) possess great potential to differentiate into multiple lineages and have self-renewal capacity, allowing them to be utilized as patient-specific cell-based therapeutics. Although the use of various stem cell-derived β-cells has been proposed as a novel approach for treating diabetes mellitus, developing an efficient method to establish highly functional β-cells remains challenging.

**Methods:**

We aimed to develop a novel cell culture platform that utilizes a fibroblast growth factor 2 (FGF2)-immobilized matrix to regulate the adhesion and differentiation of hO-MSCs into insulin-producing β-cells via cell–matrix/cell–cell interactions. In our study, we evaluated the in vitro differentiation potential of hO-MSCs cultured on an FGF2-immobilized matrix and a round-bottom plate (RBP). Further, the in vivo therapeutic efficacy of the β-cells transplanted into kidney capsules was evaluated using animal models with streptozotocin (STZ)-induced diabetes.

**Results:**

Our findings demonstrated that cells cultured on an FGF2-immobilized matrix could self-organize into insulin-producing β-cell progenitors, as evident from the upregulation of pancreatic β-cell-specific markers (PDX-1, Insulin, and Glut-2). Moreover, we observed significant upregulation of heparan sulfate proteoglycan, gap junction proteins (Cx36 and Cx43), and cell adhesion molecules (E-cadherin and Ncam1) in cells cultured on the FGF2-immobilized matrix. In addition, in vivo transplantation of differentiated β-cells into animal models of STZ-induced diabetes revealed their survival and engraftment as well as glucose-sensitive production of insulin within the host microenvironment, at over 4 weeks after transplantation.

**Conclusions:**

Our findings suggest that the FGF2-immobilized matrix can support initial cell adhesion, maturation, and glucose-stimulated insulin secretion within the host microenvironment. Such a cell culture platform can offer novel strategies to obtain functional pancreatic β-cells from patient-specific cell sources, ultimately enabling better treatment for diabetes mellitus.

**Graphical Abstract:**

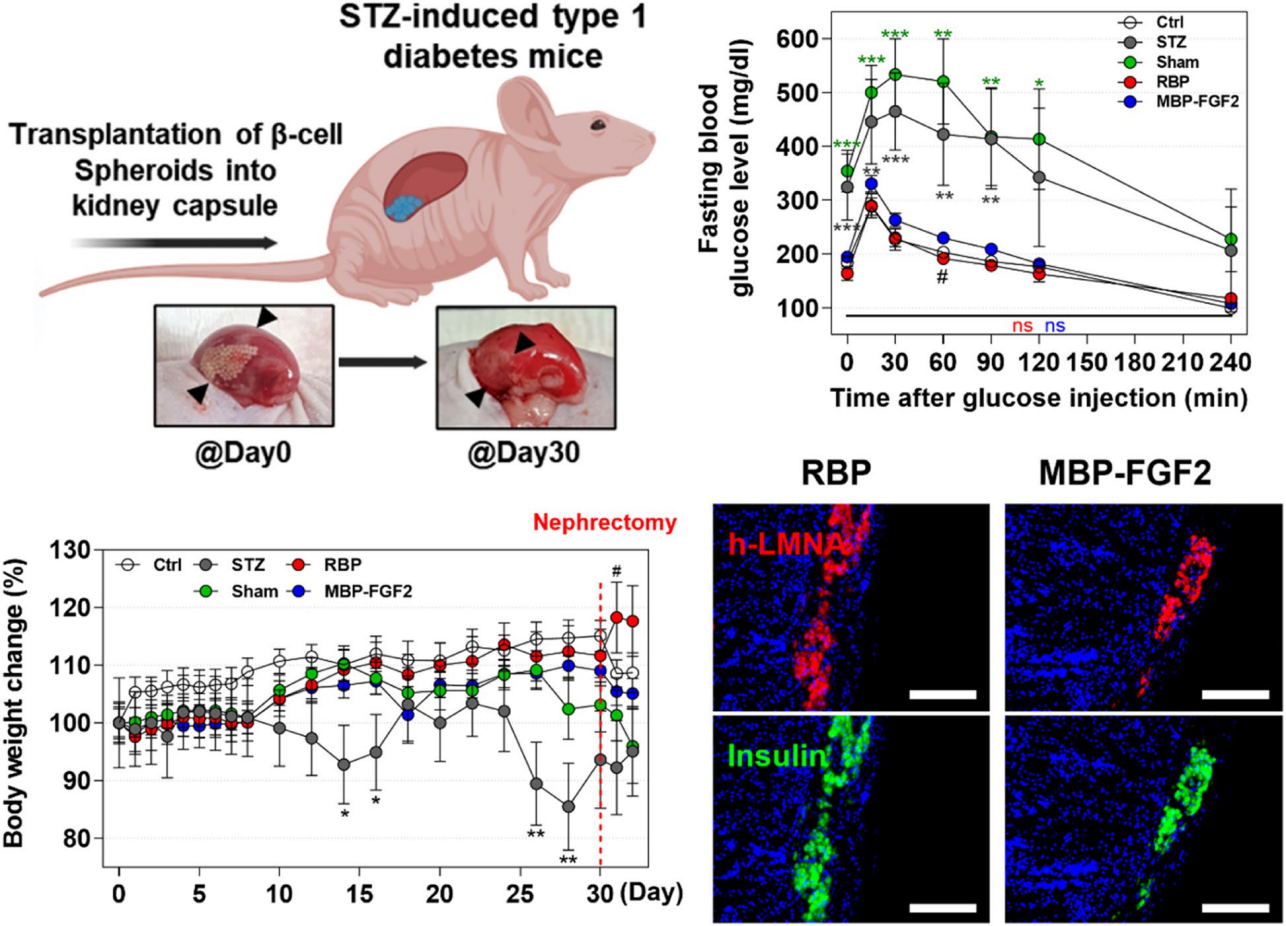

**Supplementary Information:**

The online version contains supplementary material available at 10.1186/s40824-023-00419-1.

## Background

Diabetes mellitus is a major cause of mortality worldwide and type I diabetes mellitus (T1DM) is characterized as a chronic metabolic disorder caused by autoimmune dysfunctions that destroy the mass of insulin-producing cells, known as β-cells, in the pancreas, thus inducing severe hyperglycemia [1]. As exogenous insulin is the standard therapy for T1DM, current T1DM therapies focus on controlling the blood glucose levels using exogenous insulin and promoting insulin secretion by β-cells [2]. Although exogenous insulin controls glucose levels, its use has limitations as it cannot physiologically regulate blood glucose levels; this increases the risk of hypoglycemic events and necessitates constant external monitoring. However, stem cell-based therapies have the potential to provide insulin by replacing damaged insulin-producing cells, thus negating these limitations [3, 4].

Recently, stem cell-based therapy has emerged as an alternative treatment for curing or preventing T1DM [5]. Previous studies have shown that functional human β-cells can be generated in vitro from human pluripotent stem cells (hPSCs) and these hPSC-derived β-cells function in a manner similar to primary human β-cells, demonstrating enhanced glucose-stimulated insulin secretion in diabetic mice along with reduced hyperglycemia [6–8]. Further, adult stem cells have self-renewal capacity and multilineage potential [9]. For example, mesenchymal stem cells (MSCs) are non-hematopoietic precursor cells located in various tissues, such as the adipose, dermis, and bone marrow; they can differentiate along mesodermal lineages into adipocytes, osteocytes, and chondrocytes [10–13]. Hence, hPSCs and hMSCs are ideal and promising therapeutic tools for advancing regenerative medicine [14, 15]. Previously, functional human β-cells have been generated from hMSCs such as adipose-derived MSCs and tonsil-derived MSCs [16]. MSCs can also be isolated from omentum fat [17, 18]. The characteristics of omentum-derived MSCs (O-MSC) have been shown to be similar to those of adipose-derived MSCs based on their proliferation, surface antigen expression, and differentiation potential [19, 20]. Furthermore, owing to the easy accessibility and abundant availability of omentum fat, O-MSCs have been considered as a potential source for autologous regenerative and cellular therapeutic candidates [21, 22]. Therefore, in this study, human omentum fat-derived MSCs were isolated and used as a potential autologous stem cell source for differentiation into β-cells.

The pancreas is a multicellular organ consisting of β-cells that respond to elevated blood glucose levels by secreting insulin [23]. The gap junction proteins, connexins, are related to cell–cell coupling, which contributes to maturation and insulin secretion in pancreatic cells [24, 25]. A previous study showed that 2D in vitro cultures of differentiated β-cells do not maintain a β-cell phenotype and function comparable to that of β-cells and that extracellular matrix (ECM) interactions play an important role in β-cell survival, proliferation, insulin secretion, and maintenance of 3D morphology [26, 27]. Furthermore, 3D cultures are important for mimicking the native 3D microenvironment, which is generally achieved using natural or synthetic biomaterial-based scaffolds, thereby promoting β-cell survival and function [28].

Previously, we developed a unique material for 3D cultures, which does not require a biomaterial scaffold as the culture spontaneously assembles into 3D spheroids on a maltose-binding protein-basic fibroblast growth factor (MBP-FGF2)-immobilized polystyrene plate (PS) [29, 30]. Here, we describe a new platform that controls cell–matrix interactions and promotes cell–cell interactions to create highly functionalized 3D β-cells without a biomaterial scaffold. This 3D culture platform has been shown to induce spontaneous cell aggregation and promote cell–cell interactions through cell adhesion molecules and gap junction proteins, thus promoting hO-MSC differentiation into β-cells and enhancing insulin secretion. The effect of the MBP-FGF2-immobilized surface on β-cell differentiation and insulin secretion as well as the role of hO-MSC interaction on MBP-FGF2 during differentiation was evaluated. This study further describes the cell–cell and cell–matrix interactions that promote the differentiation of hO-MSCs into β-cells on the MBP-FGF2 surface, which may provide a new platform for drug screening and cell-based therapies for T1DM.

## Materials and methods

### Isolation of hO-MSCs from human omentum tissue using magnetic activated cell sorting (MACS)

Omentum tissue was obtained from patients aged 29–55 years who underwent laparoscopic cholecystectomy for chronic cholecystitis; these patients were recruited between May 2019 and April 2020. The study was reviewed and approved by the Soonchunyang University Hospital (Bucheon, Korea) Institutional Review Board (protocol number: 2019–03-008–007) and informed consent was obtained from all participating patients before the surgery. Laparoscopic cholecystectomy was performed under general anesthesia and the surrounding omentum tissue (usually weighing approximately 1.8–6.5 g) attached to the gall bladder because of inflammatory adhesions was harvested. The harvested omentum tissue was washed with phosphate buffer saline (PBS), minced to 1 mm^3^ pieces and enzymatically digested in the DMEM-HG supplemented with 1% P/S, 10% FBS, and 1 mg/mL collagenase type I (17,100–017, Gibco, Waltham, MA, USA) for 1 h at 37 °C. After enzymatic digestion, the collagenase solution was inactivated using DMEM supplemented with 10% FBS and the isolated cells were filtered through a 100 μm cell strainer. Next, centrifugation was performed at 300 × *g* for 10 min to separate the oil and the cell pellet was then washed twice with PBS. Cells were further purified using gradient centrifugation with Ficoll (1.073 density), washed twice with PBS, and cultured in DMEM-HG supplemented with 1% P/S and 10% FBS at 37 °C in a humidified atmosphere containing 5% CO_2_, prior to MACS (Miltenyi Biotec, Kölle, Germany). To purify O-MSCs, cells were first sorted against anti-epithelial cell adhesion molecule (EpCAM) using anti-EpCAM magnetic beads (130–061-101, Miltenyi Biotec, Kölle, Germany) to acquire EpCAM-negative cells, which were further sorted against anti-fibroblast magnetic beads (130–100-132, Miltenyi Biotec, Kölle, Germany). All magnetic beads were re-suspended in buffer solution containing 0.5% w/v bovine serum albumin (BSA) and 2 mM EDTA in PBS at a dilution ratio of 1:10 in the buffer solution, and the cells were then sorted using an MS column (130–042-201, Miltenyi Biotec, Kölle, Germany).

### Flow cytometry

Isolated hO-MSCs were characterized using fluorescence-activated cell sorting (FACS). At least 100,000 isolated hO-MSCs were used for the analysis of each cell surface marker. Briefly, hO-MSCs were harvested and stained with the cell surface marker antibodies described below for 30 min at 4 °C in the dark and analyzed using a flow cytometer (FACS Canto II, Becton Dickinson and Company, Franklin Lakes, NJ, USA) at the Soonchunhyang Biomedical Research Core Facility of the Korea Basic Science Institute (KBSI). Antibodies were diluted 1:20 with FACS buffer containing 1% BSA in PBS and fluorescence-labeled cells were kept on ice during FACS analysis. MSC-positive markers CD29-PE (cat# 303,004, BioLegend, San Diego, CA, USA), CD44-FITC (cat# 103,022, BioLegend, San Diego, CA, USA), CD73-FITC (cat# 344,016, BioLegend, San Diego, CA, USA), CD105-PE (cat# 12–1057-42, eBioscience, San Diego, CA, USA), and CD166-PE (cat# 343,904, BioLegend, San Diego, CA, USA); and MSC-negative markers CD45-FITC (cat# 368,508, BioLegend, San Diego, CA, USA), CD34-FITC (cat# 343,517, BioLegend, San Diego, CA, USA), and CD31-PE (cat# 303,106, BioLegend, San Diego, CA, USA) were used for flow cytometry.

### In vitro differentiation of hO-MSCs into three mesodermal lineages

To confirm the stemness of hO-MSCs, cells were differentiated into mesodermal lineages, such as adipocytes, osteocytes, and chondrocytes. For adipogenic differentiation, cells were grown until 100% confluency and then differentiated into adipocytes using a commercially available adipogenic medium (CB-adipo-BM, CEFO Bio, Seoul, Korea). Cells were cultured for 2 weeks to achieve adipogenic differentiation and then analyzed. Following fixation with 4% paraformaldehyde, cells were stained with Oil Red O (O0625, Sigma-Aldrich, St. Louis, MO, USA) and lipidTOX (H34475, Invitrogen, Waltham, MA, USA), which specifically stain lipids in cells.

For osteogenic differentiation, cells were grown until approximately 80% confluent and then differentiated into osteocytes in a commercially available osteogenic medium (CM-DM-osteo, CEFO Bio, Seoul, Korea). Cells were cultured for 2 weeks and then analyzed. The fixed cells were stained with Alizarin Red (A5533, Sigma-Aldrich, St. Louis, MO, USA) and anti-osteocalcin antibody (OCN; B0916, Santa Cruz, Dallas, TX, USA), which specifically stain calcium and OCN.

For chondrogenic differentiation, 1 × 10^5^ cells per well were cultured on ultra-low binding round-bottom plates (RBP) to formed spheroids that were incubated with a chondrogenic medium containing low-glucose DMEM (10–014-CV, Corning, New York, NY, USA), 1% P/S, 1% ITS + premix (354,352, Corning), 100 nM dexamethasone (D2915, Sigma-Aldrich), 40 μg/mL proline (H54409, Sigma-Aldrich, St. Louis, MO, USA), 50 μg/mL ascorbic acid-2-phosphate (A8960; Sigma-Aldrich, St. Louis, MO, USA), and 10 μg/mL transforming growth factor-β1 (human recombinant TGF-β1, 30R AT027, Fitzgerald, Acton, MA, USA) for 3 weeks and then analyzed. The fixed spheroids were incubated overnight with 20% w/v sucrose solution and embedded in Optimal Cutting Temperature (OCT). The embedded samples were cut into 15 μm-thick sections using a cryostat (Leica, Wetzlar, Germany). For Alcian blue staining, which specifically stains glycosaminoglycans (GAGs), samples were incubated with Alcian blue solution (A3157, Sigma-Aldrich, St. Louis, MO, USA). For immunofluorescence of collagen type 2, cryosections were incubated with anti-collagen type II (II-II6B3-C, DSHB, Iowa City, IA, USA) antibody for 1 h at room temperature. After washing with PBS, the sections were incubated with a fluorescence-conjugated secondary antibody (Invitrogen, Carlsbad, CA, USA). All images were captured using a fluorescence microscope (Eclipse Ti-U, Nikon, Tokyo, Japan) at the Soonchunhyang Biomedical Research Core Facility of the KBSI.

### Preparation of MBP-FGF2 and the MBP-FGF2-immobilized surface

A surface with immobilized FGF2 was prepared on the hydrophobic PS surface of a 48-well-plate using a maltose-binding protein (MBP) as a physical linker [31]. Briefly, MBP-FGF2 protein was produced from *Escherichia coli* using the pMAL-bFGF plasmid, which was generated by transforming a vector plasmid (New England Biolabs, Ipswich, MA, USA) with human FGF2 complementary DNA (Bioneer, Daejeon, Korea). For immobilizing MBP-FGF2 onto the PS surface, 200 μL of 10 mg/mL MBP-FGF2 protein in sterile PBS was added to each well of the 48-well plate, incubated at room temperature for 4 h, washed with sterile PBS to remove non-immobilized MBP-FGF2 protein, and further blocked with 1% BSA (SM-BOV-100, Geneall, Seoul, Korea) for 1 h to prevent the non-specific adhesion of seeded cells onto the FGF2-immobilized surface.

### Differentiation of hO-MSCs into self-organized β-cell spheroids

Β-cell differentiation was induced using two different methods. First, 1 × 10^5^ cells were seeded on a RBP (conventional method) with DMEM-HG supplemented with 1% P/S, 10% FBS at 37 °C in an atmosphere of 5% CO_2_ for forming spheroids. Alternatively, 1 × 10^5^ cells were seeded onto the MBP-FGF2-immobilized surface and self-organized into spheroids after 48 h. Spheroids formed using both methods were further cultured with β-cell differentiation media containing DMEM/F12 (11,320–033, Gibco, Waltham, MA, USA), 1% P/S, B27 (A3582801, Thermo Fisher Scientific, Waltham, MA, USA), and N2 supplement (17,502,048, Thermo Fisher Scientific, Waltham, MA, USA). To induce β-cell differentiation, 50 ng/mL of activin A (HZ-1138, Proteintech, Chicago, IL, USA) and 2 mM valproic acid (P4543, Sigma-Aldrich) were added for days 0 to 3. Next, on days 3–6, 10 nM exendin-4 (E7144, Sigma-Aldrich, St. Louis, MO, USA) and 10 ng/mL FGF2 (100-18B, PeproTech, East Windsor, NJ, USA) were added. Finally, on days 6–15, 50 ng/mL exendin-4 and 10 mM nicotinamide were added. The medium was changed every 3 days.

### Immunocytochemistry

Fixed and cryosectioned samples were blocked with 3% (w/v) BSA in PBS and permeabilized with 0.3% v/v Triton X-100 for 1 h at room temperature. Samples were incubated with the following primary antibodies in 1% w/v BSA for 1 h at room temperature: mouse anti-osteocalcin (1:200; cat#, Santa Cruz Biotechnology, Santa Cruz, CA, USA), mouse anti-collagen type II (1:200; II-II6B3-C, DSHB), goat anti-PDX-1 (1:200; 47,383, Abcam, Cambridge, UK), guinea pig anti-insulin (1:200; ab7842, Abcam, Cambridge, UK), and rabbit anti-connexin 36 (1:200; 364,600, Invitrogen, Carlsbad, CA, USA). Samples were washed with PBS and incubated with the following secondary antibodies: anti-rabbit Alexa 555 (1:200; A21428, Thermo Fisher Scientific, Waltham, MA, USA), anti-rabbit Alexa 647 (1:200; cat#, Thermo Fisher Scientific, Waltham, MA, USA), anti-mouse Alexa 488 (1:200; A11001, Thermo Fisher Scientific, Waltham, MA, USA), anti-guinea pig Alexa 488 (1:200; A11073, Thermo Fisher Scientific, Waltham, MA, USA), and anti-goat Alexa 555 (1:200; A31573, Thermo Fisher Scientific, Waltham, MA, USA). Nuclei were stained with 2 mg/mL Hoechst 33,342 (1:1000; H21492; Molecular Probes, Eugene, CA, USA) for 10 min at room temperature. Fluorescence images were acquired using a confocal microscope (LSM 710; Carl Zeiss, Oberkochen, Germany) at the Soonchunhyang Biomedical Research Core Facility of the KBSI.

### Quantitative RT-PCR analysis

Total RNA was extracted using TRIzol reagent (Invitrogen, Carlsbad, CA, USA) and reverse transcribed using ReverTra Ace qPCR RT master mix with gDNA Remover (Toyobo, Osaka, Japan) according to the manufacturer’s protocol. qPCR was performed using SYBR Green Real-time PCR master mix (Toyobo) on a QuantStudio Real-Time PCR system (Applied Biosystems, Foster City, CA, USA) and the target gene expression was calculated using the 2^−ΔΔCt^ method with glyceraldehyde-3-phosphate dehydrogenase (GAPDH) as the reference gene [32]. The target genes and their primer sequences are provided in Supplementary Table S1.

### Glucose-stimulated insulin secretion (GSIS) analysis

Glucose-stimulated insulin secretion (GSIS) analysis was performed to evaluate the glucose-sensitive insulin secretion ability of β-cell spheroids (1 × 10^5^ cells / spheroid). β-cell spheroids (48 spheroids) were washed twice with D-PBS and pre-incubated in 500 µL of basal glucose (2.8 mM; d-glucose, G8270, Sigma-Aldrich, St. Louis, MO, USA) for 1 h at 37 °C in a humidified atmosphere containing 5% CO_2_ in Krebs–Ringer Bicarbonate HEPES buffer (KRBH) containing 129 mM NaCl, 5 mM NaHCO_3_, 4.8 mM KCl, 1.2 mM KH_2_PO_4_, 1.0 mM CaCl_2_, 10 mM HEPES (pH 7.4), and 0.1% w/v BSA. After 1 h, these β-cell spheroids were incubated in both low-glucose (2.8 mM glucose in KRBH buffer) and high-glucose (16.7 mM glucose in KRBH buffer) solutions. The supernatant of cells cultured under the two different glucose concentrations, which contained insulin secreted by β-cell spheroids, was collected. Subsequently, β-cell spheroids were lysed using a lysis solution (77% v/v absolute ethanol and 1% v/v 12 N hydrochloric acid in DI water). The cell-secreted insulin and insulin within β-cell spheroids were quantified using a human insulin enzyme-linked immunosorbent assay (ELISA) (ab100578, Abcam, Cambridge, UK) according to the manufacturer’s instructions. Insulin secretion is presented as a percentage of the total cellular insulin content, which was calculated as insulin levels normalized to the total cellular insulin content in both basal and stimulated secretions, as well as in the cell extracts. Additionally, the stimulation index (SI) was calculated as the ratio of insulin secretion during incubation with stimulatory glucose (16.7 mM) to that with basal glucose (2.8 mM) [33].

### Animal models of STZ-induced diabetes

STZ was dissolved in cold 50 mM sodium citrate buffer at pH 4.5 immediately prior to use. Before STZ injection, mice were fasted for 16 h; 8-week-old male athymic nude mice (J:NU, #007859, Jackson Laboratories) were injected intraperitoneally with a low dose of 50 mg/kg streptozotocin (STZ, S0130-500MG, Sigma-Aldrich, St. Louis, MO, USA) for three consecutive days [34]. Diabetes was confirmed when non-fasting blood glucose levels exceeded 400 mg/dL, and mice were then randomly assigned to the experimental groups. All animal studies were approved by the Institutional Animal Care and Use Committee (IACUC) at the Soonchunhyang University (protocol number: SCH22-0013).

### Transplantation of insulin-producing β-cells into animal models of STZ-induced diabetes

In our study, we transplanted β-cell spheroids into the kidney capsule, an extravascular site known to offer a semi-permeable barrier, to facilitate the survival and engraftment of transplanted cells, according to previously described protocols [35, 36]. Briefly, 120 hO-MSC-derived β-cell spheroids (1 × 10^5^ cells / spheroid) were collected in polyethylene tubes (inside diameter: 0.58 mm and outside diameter: 0.965 mm) connected to a 1 mL syringe. Mice with STZ-induced diabetes were anesthetized with 3–4% isoflurane (Hana pharm, Seongnam-si, Korea). Incisions were made in the skin to expose the kidneys, and the subcapsular membrane of the kidney was carefully opened using the tip of a needle to allow the entry of the β-cell spheroids-containing polyethylene tube. Non-transplanted (Ctrl and STZ group), PBS-injected (sham group), and 120 β-cell spheroid-transplanted (RBP and MBP-FGF2 groups) mice were subjected to the respective treatment in the kidney capsule. The subcapsular membrane of the kidney was then closed using bipolar electrosurgery (Sammi-medical, Seoul, Korea), and the kidney was returned to the original location. After suturing the skin, the mice were placed on a heating pad. Non-fasting blood glucose levels were measured daily until day 7, and every 2 days until day 30 (values greater than 600 mg/dL were recorded as “HI” and assigned a value of 600 mg/dL). In addition, body weight was measured, and all blood glucose measurements were taken in the early morning in a fed state.

After 30 days, the mice were anesthetized with isoflurane to remove the kidney containing the transplanted β-cell spheroids (nephrectomy). The skin was incised to expose the kidney, and the blood vessels directly connected to the kidney were cut using bipolar electrosurgery to prevent bleeding. The kidneys were then fixed with 4% w/v PFA in PBS for histology. After the skin was sutured, the mice were placed on a heating pad. Non-fasting blood glucose levels and weight were measured for 2 consecutive days after nephrectomy.

### Intraperitoneal glucose tolerance test (IPGTT)

The fasting blood glucose levels of mice were measured after 16 h of fasting, prior to the intraperitoneal injection of glucose. Briefly, a stock solution of 10% w/v glucose dissolved in PBS was prepared for the injection at a dosage of 1 g/kg body weight. Blood glucose levels were measured at 15, 30, 60, 90, 120, and 240 min after the injection.

### Quantification of mouse and human insulin in plasma using ELISA

Mouse and human insulin levels were quantified in plasma collected from mice with STZ-induced diabetes using ELISA kits specific for each species (mouse: 90,082, Crystal Chem, IL, USA; human: ab100578, Abcam, Cambridge, UK). Blood samples were collected from the tail vein in a non-fasting state at 15 days and 30 days using mini-vettes (50 μl, POCT, Nümbrecht, Germany). The plasma was obtained by incubating the blood for 1 h on ice, followed by centrifugation at 3000 g for 20 min; the separated plasma was then transferred to a new tube. The amounts of insulin in the plasma were quantified using the ELISA kit according to the manufacturer’s instructions.

### Histological analysis of kidney tissues

To validate the therapeutic potential of transplanted β-cell spheroids within animal models of STZ-induced diabetes, the kidney was fixed in 4% PFA for 1 day, embedded in OCT blocks, and sectioned at a thickness of 8 μm. The samples were then cryosectioned and incubated in DI water for 10 min, followed by blocking with 20% v/v normal goat serum (S-1000, Vector Laboratories, Mowry Ave, CA, USA) in PBS for 1 h. Next, the samples were incubated with the following primary antibodies for 1 h at room temperature: guinea pig anti-insulin (1:200; ab7842, Abcam, Cambridge, UK) and rabbit anti-human-specific lamin A/C (1:200, ab108595, Abcam, Cambridge, UK). The samples were then washed with PBS and incubated with the following secondary antibodies: anti-rabbit Alexa Fluor® 555 (1:200; A21428, Thermo Fisher Scientific, Waltham, MA, USA), anti-guinea pig Alexa Fluor® 488 (1:200; A11073, Thermo Fisher Scientific, Waltham, MA, USA) to visualize the bound primary antibodies. Nuclei were counterstained with 2 mg/mL Hoechst 33,342 (1:1000; H21492; Molecular Probes, Eugene, CA, USA) for 10 min at room temperature. For hematoxylin and eosin (H&E) staining, samples were incubated with hematoxylin (MHS16, Sigma-Aldrich, St. Louis, MO, USA) and eosin (MA0101015, BBC Biochemical, Mount Vernon WA, USA). Fluorescence and bright field images were acquired using a microscope (Evos M7000, Invitrogen, Carlsbad, CA, USA) at the Soonchunhyang Biomedical Research Core Facility of the Korea Basic Science Institute.

### Statistical analysis

All values are presented as the mean ± standard error of the mean of three replicates. Statistical significance was assessed using one-way analysis of variance (ANOVA) with Tukey’s multiple comparison tests in GraphPad Prism 9.0 (GraphPad, San Diego, CA, USA) software. Statistical significance was set at *p < 0.05, **p < 0.01, and ***p < 0.001.

## Results

### Isolation and characterization of hO-MSCs

**Fig. 1:**
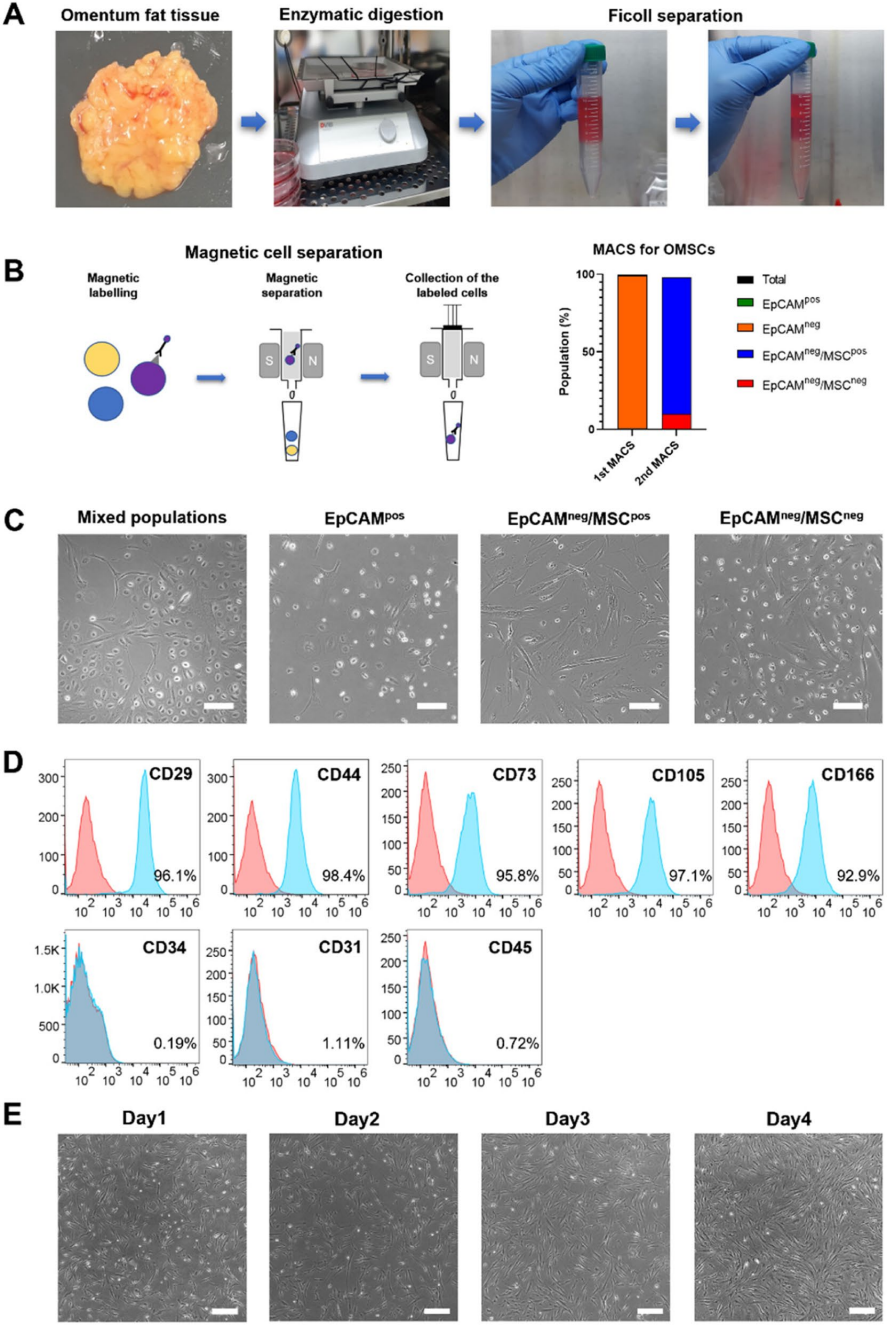
Isolation and characterization of human omentum-derived MSCs (hO-MSCs). **A** Isolation of hO-MSCs. **B** Magnetic cell separation and percentage of cell population. **C** Images of separated hO-MSCs (scale bar = 200 μm). **D** Flow cytometry analysis of hO-MSCs using MSC-positive and -negative cell surface markers. **E** Time-dependent culture images of hO-MSCs during proliferation after magnetic cell separation (scale bar = 400 μm)

As shown in Fig. 1A, omentum tissue-derived cells were isolated through enzymatic digestion and density-based Ficoll separation; these cells morphologically exhibited two phenotypes, cuboidal and spindle-shaped. Thus, to purify hO-MSCs, MACS was conducted against anti-EpCAM and anti-fibroblast magnetic beads (Fig. 1B-C). After separation using anti-EpCAM magnetic beads, the cells still showed two distinct populations. Ultimately, EpCAM-negative cell populations were further sorted using anti-fibroblast magnetic beads. As a result, the omentum-derived cells showed a 99.2% EpCAM-negative population (1^st^ MACS), and among these, approximately 88% of the cells exhibited both EpCAM-negative and fibroblast-positive population (hO-MSCs). Following MACS separation, cells were observed to be mesenchymal-shaped and attached to the cell culture dishes (Fig. 1B-C). To characterize the MSC surface marker expression of hO-MSCs, we expanded them and analyzed their MSC surface marker expression using flow cytometry (Fig. 1D). The hO-MSCs expressed CD29, CD44, CD73, CD105, and CD166, which are characteristically expressed in MSCs. In contrast, hO-MSCs did not express CD45, CD34, and CD31, which are expressed on epithelial cells, but not in MSCs [37, 38]. The isolated hO-MSCs showed cellular homogeneity, a fibroblast shape, and exhibited proliferative ability (Fig. 1E). Additionally, to confirm their multilineage differentiation potential, we demonstrated that these hO-MSCs could differentiate into adipocytes, osteocytes, and chondrocytes (Supplementary Fig. S1). Overall, these results suggest that the MACS-sorted cells used in this study were indeed MSCs with potential for differentiation.

### 3D spheroid formation and differentiation of hO-MSCs into β-cells on an MBP-FGF2 immobilized surface

**Fig. 2:**
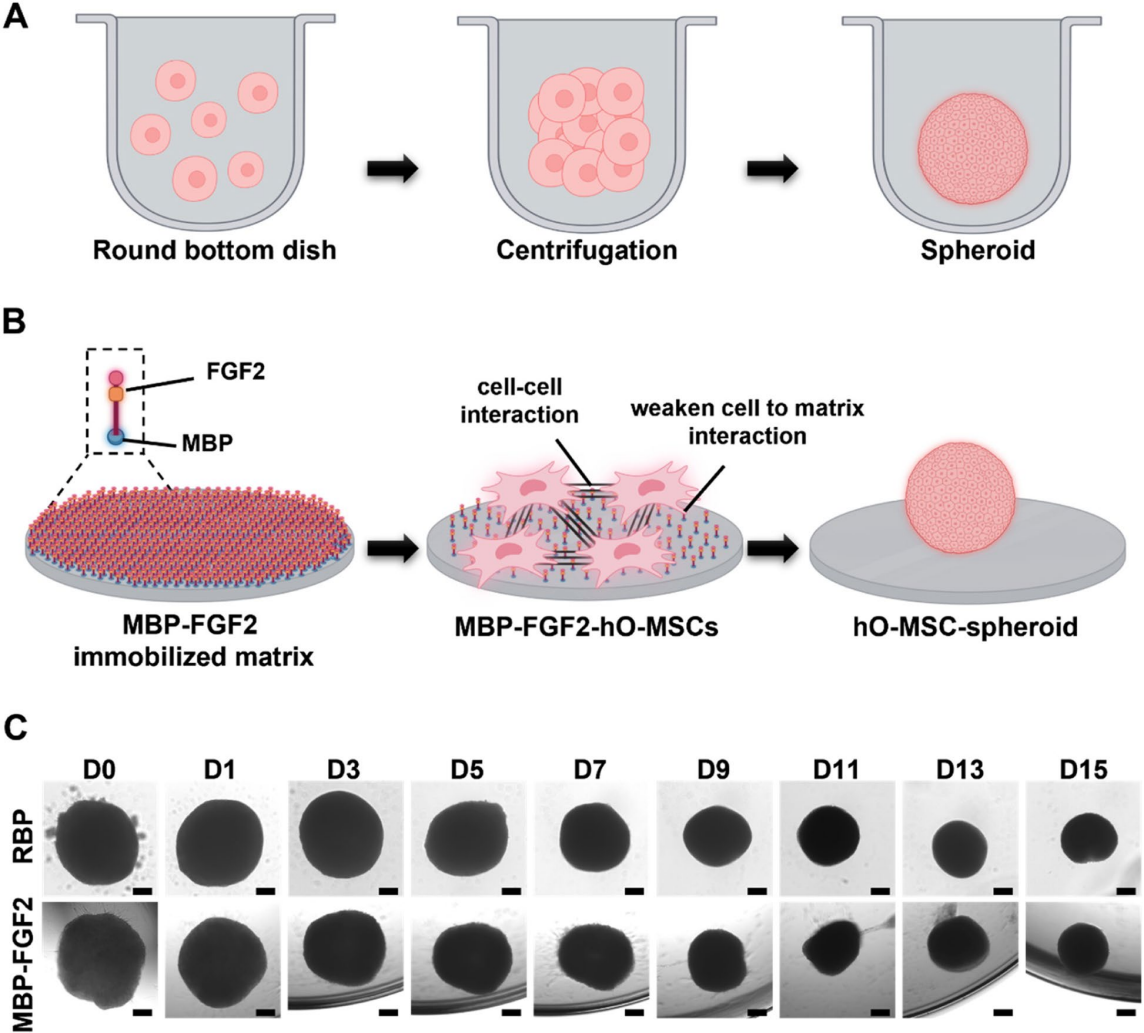
Differentiation of hO-MSCs into β-cells using two different culture methods: round-bottom plate (RBP) and maltose-binding protein-basic fibroblast growth factor 2 (MBP-FGF2) immobilized surface). **A** Schematic illustration of the RBP and **B** MBP-FGF2 cell culture methods (created with BioRender.com). **C** Phase contrast images of hO-MSC spheroids during β-cell differentiation using different culture methods. Scale bar = 200 μm

Next, we coated a PS plate surface with MBP-FGF2 protein to promote β-cell differentiation and maturation. MBP contains a hydrophobic domain that can physically link with the PS surface; the adsorption behavior of MBP-FGF2 on the PS surface was examined using quartz crystal microbalance analysis as well as heparin binding and cell adhesion assays [39, 40]. The hO-MSCs were cultured and differentiated into β-cells on two types of culture platforms, round-bottom plates (RBP) and MBP-FGF2 immobilized surfaces, to investigate the differences in β-cell differentiation under these two culture conditions (Fig. 2A). In the RBP group, 1 × 10^5^ hO-MSCs/well were seeded on a RBP and centrifuged to induce cell aggregation. Alternatively, 1 × 10^5^ hO-MSCs/well were seeded on an MBP-FGF2 immobilized surface. Unlike suppression of cell surface adhesion observed in cells cultured on the RBP plate, hO-MSCs attached to the MBP-FGF2 immobilized surface after seeding for 4 h. After 12 h, hO-MSCs detached and rolled from the edge of the plate and at 48 h after seeding, the hO-MSCs underwent further condensation and spontaneously assembled uniformly into 3D spheroids (data not shown). The behavior of hO-MSC spheroids was monitored on both surfaces during β-cell differentiation (Fig. 2B). On both surfaces, hO-MSCs condensed into 3D spheroids within 48 h and maintained an intact 3D spheroid structure during long-term differentiation. Our results thus suggested that the immobilized MBP-bFG2 supported hO-MSC adhesion and allowed self-assembly into 3D spheroids that maintained the spheroid shape until the end of differentiation.

### MBP-FGF2 surface promoted β-cell differentiation

**Fig. 3:**
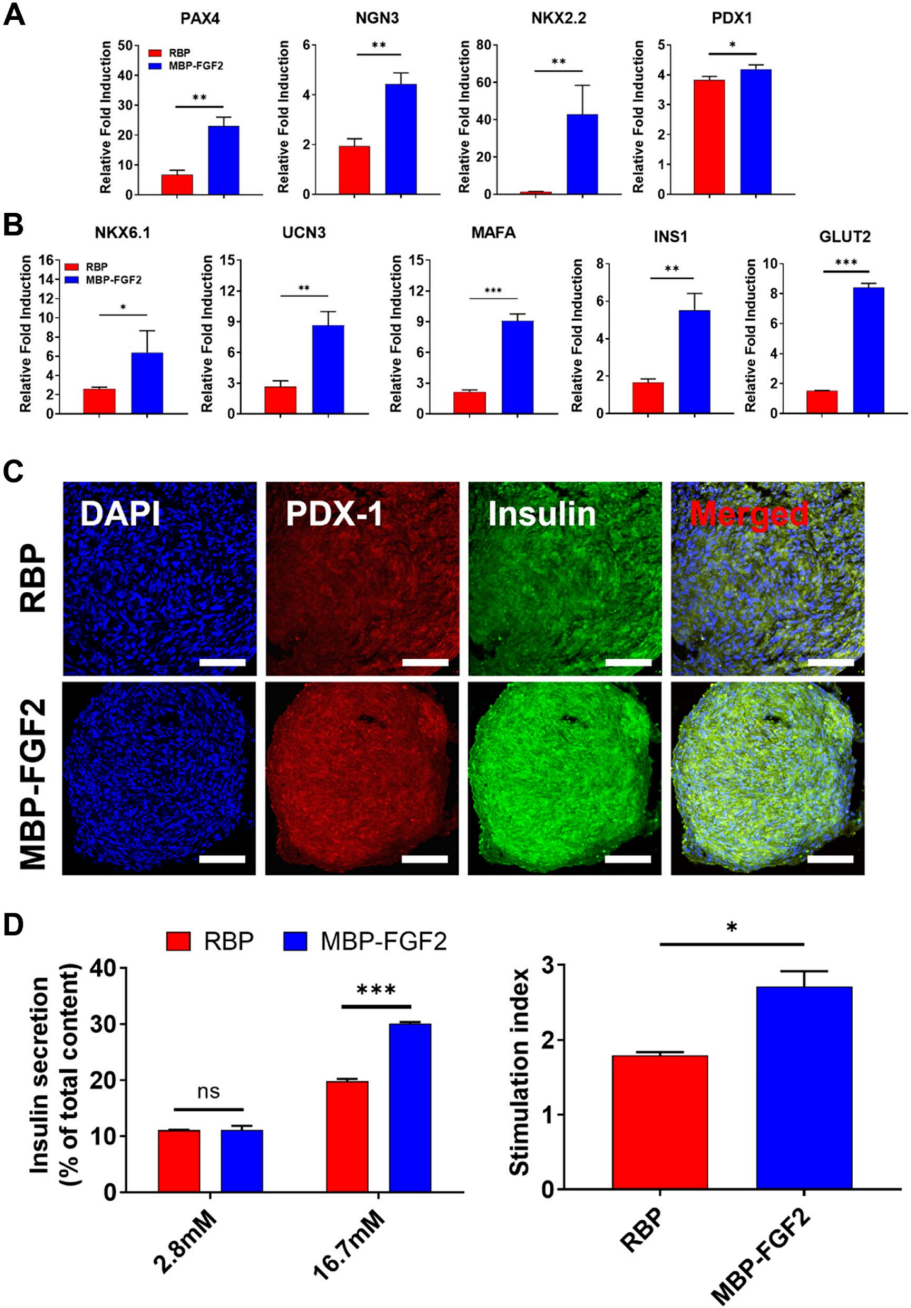
Analysis of β-cell markers and insulin secretion. **A** Gene expression of β-cell progenitor markers. **B** Transcript-level expression of β-cell maturation markers. **C** Immunofluorescence staining images of DAPI (blue), PDX-1 (red), and insulin (green). **D** Analysis of glucose-stimulated insulin secretion in low (2.8 mM) and high-glucose (16.7 mM) conditions. Insulin secretion was calculated by dividing the insulin level in the low- and high-glucose solutions by the total cell lysis insulin level. The insulin secretion index was calculated by dividing the insulin level of high-glucose by the insulin level of low-glucose. Data represent the mean ± SD, *p < 0.05; **p < 0.01; ***p < 0.001. “ns” indicates statistically non-significant. Scale bar = 100 μm. RBP, round-bottom plate; MBP-FGF2, maltose-binding protein-basic fibroblast growth factor 2

Further, we investigated the differentiation of hO-MSCs into insulin-secreting β-cells using the two types of differentiation platforms described above. These two types of platforms facilitated the formation of 3D spheroids and their differentiation into insulin-secreting β-cell spheroids. To investigate β-cell differentiation potential in both groups, gene expression analyses were conducted using qRT-PCR. As shown in Fig. 3A, we observed that hO-MSCs cultured on the MBP-FGF2 surface had higher expression levels of endocrine progenitor marker genes, such as PAX4, NGN3, Nkx2.2, and PDX-1 than those observed in hO-MSCs cultured on RBP. Additionally, when we further investigated the expression of genes related to β-cell maturation, which is crucial for insulin secretion, we observed significant upregulation of β-cell maturation-related genes, including Nkx6.1, Ucn3, and MAFA, in the cells cultured on MBP-FGF2. Moreover, in the MBP-FGF2 group, INS1 gene expression level, which provides instructions for producing insulin, was significantly 3.3-fold higher than that observed in the RBP group, while the expression level of GLUT2, a glucose-sensitive gene that is required for GSIS, was significantly 5.6-fold higher in the MBP-FGF2 group than in the RBP group (Fig. 3B). PDX1 and insulin protein expression was further investigated using immunofluorescence (Fig. 3C). Although the spheroids expressed PDX1 and insulin in both RBP and MBP-FGF2, higher levels of these proteins were observed in MBP-FGF2 compared with those in RBP. Next, to assess whether the MBP-FGF2 surface β-cell differentiation platform promoted insulin secretion in a glucose concentration-dependent manner, GSIS was used and the results were compared with those obtained after culturing on the RBP platform using a human insulin ELISA kit (Fig. 3D). Both the RBP and MBP-FGF2 groups at the end of the β-cell differentiation phase were exposed to low (2.8 mM) or high (16.7 mM) glucose concentrations in KRBH buffer. Insulin secretion was then calculated by dividing the insulin level in the low- and high-glucose solutions by the insulin level obtained after total cell lysis. Insulin levels in the low-glucose solution were not significantly different between the two culture conditions. However, in the high-glucose solution, the MBP-FGF2 β-cell spheroid group secreted a higher amount of insulin than that secreted by the RBP β-cell spheroid group. The insulin secretion index was calculated by dividing the insulin level after exposure to the high-glucose solution by the insulin level after exposure to the low-glucose solution. The insulin secretion indices were 1.79- and 2.71-fold for RBP and MBP-FGF2, respectively, demonstrating a higher insulin secretion index in the MBP-FGF2 group than that in the RBP group. These results suggest that the immobilized MBP-FGF2 surface promoted the differentiation and maturation of hO-MSCs into β-cells, in addition to GSIS, as demonstrated by the expression of β-cell progenitors and maturation marker genes and the quantification of secreted insulin.

### MBP-FGF2 upregulated intercellular junction proteins via FGF2-SDC4 and TGF-βR3 interactions

**Fig. 4:**
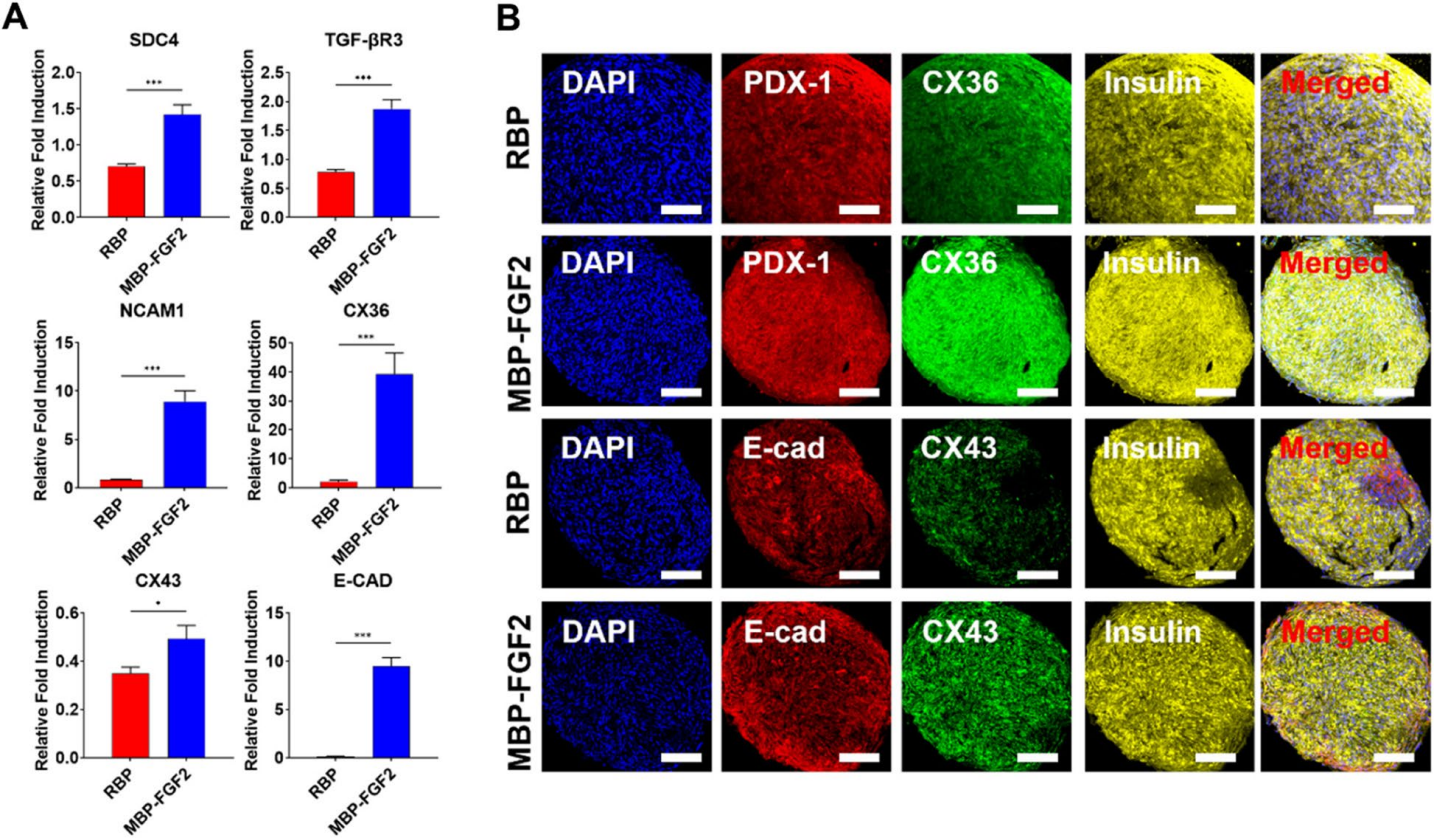
Analysis of cell surface receptors (SDC4 and TGF-βR3) and cell–cell junctions related to insulin secretion by β-cells (NCAM1, CX36, CX43, and E-cad). **A** Gene expression of cell surface receptors and cell–cell junctions related to insulin secretion by β-cells. **B** Immunofluorescence staining images of DAPI (blue), PDX-1 and E-cad (red), Cx36 and Cx43 (green), and insulin (yellow). Data represent the mean ± SD, *p < 0.05; ***p < 0.001. Scale bar = 200 μm. RBP, round-bottom plate; MBP-FGF2, maltose-binding protein-basic fibroblast growth factor 2

**Fig. 5:**
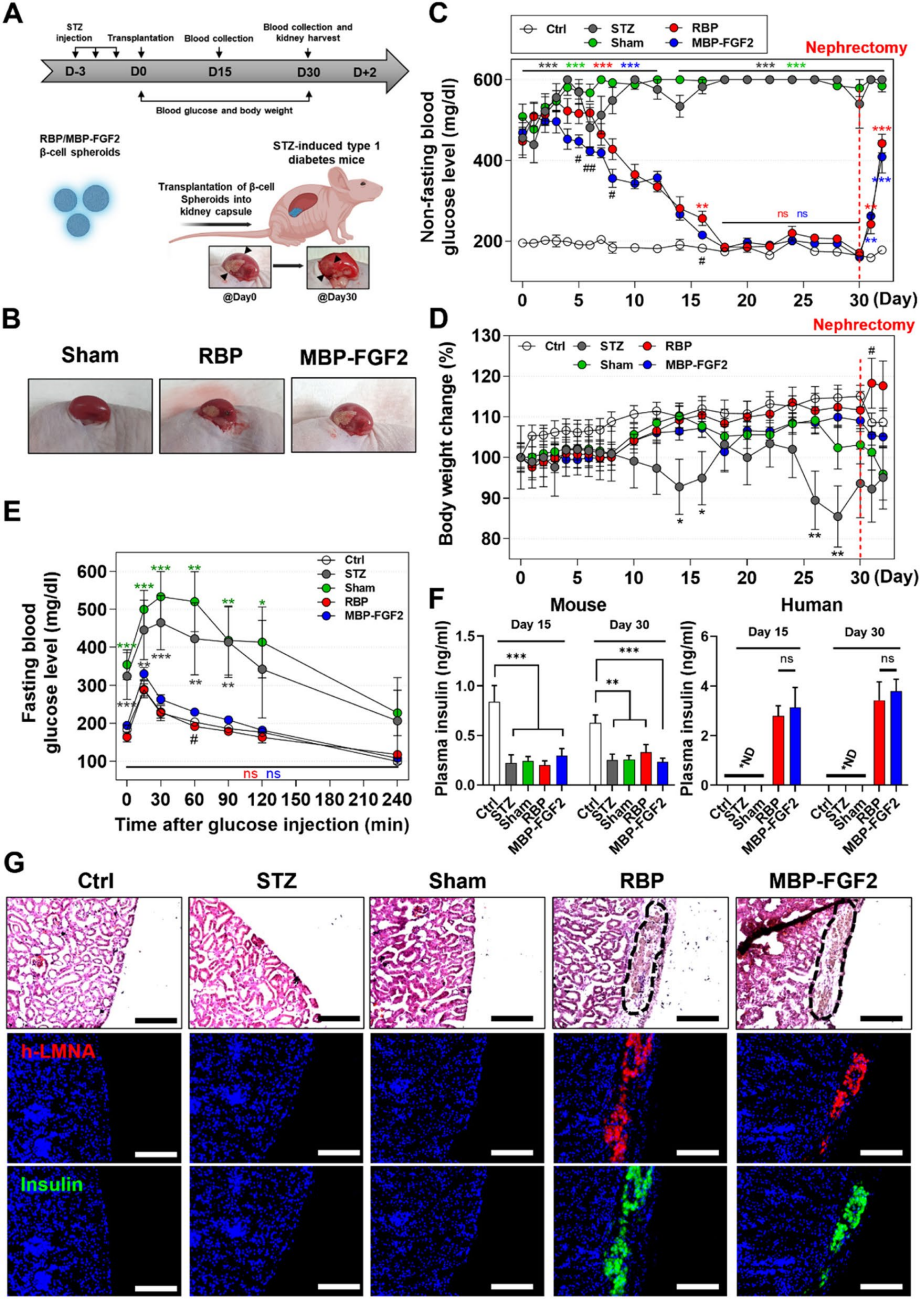
Therapeutic potential of hO-MSC-derived β-cells in an animal model of STZ-induced diabetes. **A** Schematic of experimental plan (created with BioRender.com). **B** Gross images of the kidney exhibiting transplanted β-cells within the subcapsular membrane of the kidney. **C** and **D** Non-fasting blood glucose levels and body weight were measured in streptozotocin (STZ)-induced diabetic animal models after β-cell transplantation into kidney capsules. **E** Fasting blood glucose levels were measured in animal models of STZ-induced diabetes after glucose injection at a dosage of 1 g / kg body weight. **F** ELISA of mouse and human insulin in mouse plasma after 15 and 30 days of β-cell transplantation (“ND” indicates “no detection”). **G** Immunofluorescence staining images of DAPI (blue), human-specific lamin A/C (LMNA) (red), and insulin (green). Scale bar = 150 μm. Ctrl (*n* = 5), STZ (*n* = 5), Sham (*n* = 7), RBP (*n* = 8), MBP-FGF2 (*n* = 8). Data represent the mean ± SEM, *p < 0.05; **p < 0.01; ***p < 0.001 comparing the mean of each group with the mean of the control (Ctrl: white, STZ: gray, Sham: green, RBP: red, MBP-FGF2: blue). #*p* < 0.05; ##*p* < 0.01 comparing the mean of the RBP group with the mean of the MBP-FGF2 group.“ns” indicates statistically non-significant. RBP, round-bottom plate; MBP-FGF2, maltose-binding protein-basic fibroblast growth factor 2

Next, we investigated the interactions of cell-MBP-FGF2 and intracellular junctions, such as heparan sulfate proteoglycan, gap junction proteins (Cx36, and Cx43), and cell adhesion molecules (E-cadherin and Ncam1), which promote maturation and insulin secretion in the pancreatic niche environment. Cell surface adhesion proteins play a role in islet development and promote insulin secretion via intra-islet communication. To investigate the interactions between cells as well as the hO-MSC-MBP-FGF2 immobilized surface interaction, gene and protein expression analyses were conducted using qRT-PCR and immunofluorescence, respectively; the results are shown in Fig. 5. First, we observed that hO-MSCs interacting with the MBP-FGF2 surface showed increased expression levels of heparan sulfate proteoglycans, such as SDC4 and TGF-βR3, which have the affinity to bind with FGF2, compared those in hO-MSCs interacting with RBPs. FGF2 can interact with cells through cell surface proteins such as SDC4 and stimulate cell clustering via cell interactions relying on Ncam1, Cx36, Cx43, and E-cadherin, which contribute to maturation and insulin secretion in the pancreas and can increase cell–cell interactions. We observed that Ncam1, Cx36, Cx43, and E-cadherin were expressed at significantly higher levels in cells differentiated into β-cells on the MBP-FGF2 surface than in those differentiated into β-cells on RBP (Fig. 4A). Next, we validated the findings from qRT-PCR analysis, which demonstrated that the MBP-FGF2 surface promotes cell–cell interactions via cell–matrix interactions between FGF2 and the cell surface proteins, SDC4 and TGF-βR3. Further, protein expression was investigated by immunofluorescence, and the cells cultured on the MBP-FGF2 surface exhibited enhanced expression of various proteins associated with cell–cell interactions, such as Cx36, Cx43, and E-cadherin. Cell–cell interaction proteins, including Cx36, Cx43, and E-cadherin, were localized in smaller regions of cells cultured on RBP than in cells cultured on the MBP-FGF2 surface. Alternatively, in MBP-FGF2-cultured cells, the cell–cell interaction proteins were distributed throughout the spheroids, with numerous cells co-expressing insulin and PDX-1 (Fig. 4B). These results showed that cells interacted with the MBP-FGF2 surface through cell surface proteins such as SDC4 and TGF-βR3. Furthermore, the cell–matrix interactions between cell surface proteins and MBP-FGF2 enhanced Cx36-, Cx43-, E-cadherin-, and Ncam1-mediated cell–cell interactions, whereas the increased cell–cell interactions enhanced β-cell differentiation and insulin secretion.

### Transplantation of hO-MSC-derived β-cells into the kidney capsule of animal models of STZ-induced diabetes

To further investigate the therapeutic efficacy of hO-MSC-derived β-cells in regulating the blood glucose levels in animal models of STZ-induced diabetes, we transplanted 120 differentiated β-cell spheroids into the kidney capsule of these mice and monitored their blood glucose and body weight for 30 days (Fig. 5A). The β-cell spheroids formed from RBP and MBP-FGF2 groups were successfully transplanted into the kidney capsule and maintained their position without protruding out of the kidney. For the sham group, PBS was injected into the kidney capsule (Fig. 5B). As shown in Fig. 5C, the control (Ctrl) group showed normoglycemic levels, whereas both the STZ and Sham groups exhibited hyperglycemia in their non-fasting blood glucose levels and maintained hyperglycemia for 30 days. However, transplantation of β-cell spheroids formed from RBP and MBP-FGF2 efficiently decreased the blood glucose levels from hyperglycemic to normoglycemic levels and maintained their blood glucose levels for up to 30 days. More interestingly, blood glucose levels in self-assembled β-cell spheroids from the MBP-FGF2 group were significantly lower than those in the RBP group from day 4 to day 10 after transplantation. To demonstrate that the regulation of blood glucose levels was solely attributed to the transplanted β-cell spheroids, the kidneys transplanted with β-cell spheroids were surgically removed (nephrectomy), and blood glucose levels were monitored for 2 days. After nephrectomy of the kidney containing β-cell spheroids, the blood glucose levels in both RBP and MBP-FGF2 groups were elevated to a hyperglycemic condition, indicating that the regulation of blood glucose levels in mice with STZ-induced diabetes was indeed regulated by the transplanted β-cell spheroids in both the RBP and MBP-FGF2 groups (Fig. 5C). Additionally, the weight of mice in both the RBP and MBP-FGF2 groups increased, whereas the mice in both the STZ and Sham groups lost weight (Fig. 5D and Supplementary Fig. S2). In order to assess the in vivo glucose homeostasis and β-cell function, an IPGTT assay was conducted in all groups after 16 h of starvation, as shown in Fig. 5E. Despite starvation, the blood glucose levels remained significantly higher in the STZ and Sham groups compared to those in the Ctrl and transplanted β-cell spheroids groups (both RBP and MBP-FGF2 groups). Additionally, peak blood glucose levels of the STZ and Sham groups were increased up to 534 ± 94 mg/dL and 465 ± 101 mg/dL for the initial 30 min after glucose injection, and were gradually decreased to 228 ± 85 mg/dL and 207 ± 161 mg/dL after 240 min. In contrast, the peak blood glucose levels of the Ctrl and β-cell spheroid-transplanted groups (both RBP and MBP-FGF2 groups) were increased only to 288 ± 32, 290 ± 54, and 331 ± 32 mg/dL for the initial 15 min after glucose injection, and were decreased rapidly from 15 min. At 240 min, no significant difference in glucose clearance was observed among the Ctrl, RBP and MBP-FGF2 groups. To demonstrate that blood glucose levels were regulated via insulin secreted by transplanted β-cells, we measured the amount of mouse and human insulin in the plasma at 15 and 30 days after transplantation. As shown in Fig. 5F, we clearly observed that the amount of mouse insulin was decreased in mice with STZ-induced diabetes compared to that in the healthy mice (Ctrl group). Additionally, human insulin was only detected in the plasma of β-cell transplanted mice (RBP and MBP-FGF2 groups), and there was no significant difference in the insulin levels between the RBP and MBP-FGF2 groups, whereas human insulin was not detected in the non-β-cell transplanted mice (Ctrl, STZ, and Sham groups). In addition, we conducted histological evaluations to further evaluate the engraftment of transplanted β-cells. After 30 days of β-cell spheroid transplantation, the spheroids were observed to have been fully engrafted into the host tissue; we observed the formation of blood vessels around β-cell spheroids, indicating their integration with the host tissue (Supplementary Fig. S3A). As shown in Fig. 5G and Supplementary Fig. S3 B-C, H&E staining and co-expression of human-specific lamin A/C with insulin confirmed the survival and functional engraftment of transplanted β-cell spheroids from both the RBP and MBP-FGF2 groups. Taken together, these in vivo results suggest that transplantation of differentiated β-cell spheroids effectively decreased blood glucose levels from hyperglycemic to normoglycemic levels, and this therapeutic effect was sustained for up to 30 days. Furthermore, β-cell spheroids cultured on the MBP-FGF2 group exhibited significantly faster and more efficient blood glucose regulation, compared to β-cell spheroids cultured on the RBP group.

## Discussion

In T1DM, the current therapies focus on controlling blood glucose levels using exogenous insulin and promoting insulin secretion from β-cells. However, these therapies cannot overcome the abnormal function of β cells. Therefore, stem cell-based therapies are important as they aim to replace the impaired β-cells [3]. Stem cells are considered promising therapeutics for multiple disorders as they can be isolated from various patient-derived tissues, such as adipose tissue and bone marrow, both of which have been shown to possess potential for differentiating into multiple mesodermal lineages [11]. Previously, Pagliuca et al. showed that functional human β-cells can be directly generated from hPSCs in vitro and that these hPSC-derived β-cells function similarly to primary human β-cells [8]. Further, functional human β-cells can differentiate from various hMSCs, such as adipose-derived, tonsil-derived, and bone marrow-derive MSCs [16, 41]. In this study, owing to their relatively easy accessibility, scalability, ease of culture, and multilineage differentiation capacities, we utilized hO-MSCs and induced them to differentiate into β-cells, demonstrating their therapeutic potential as autologous stem cell sources [42].

FGF2 has an affinity for FGF as well as HSPG receptors on the cell surface, thus forming the FGF2-FGFR-HSPG complex and resulting in a complex innate signaling cascade [43]. Furthermore, previous studies have demonstrated that FGFR1 promotes PDX-1 expression in the early developmental stages of pancreatic progenitors, indicating that FGFR1 signaling is important during pancreatic development, β-cell survival, and β-cell proliferation [44, 45]. Previously, we had developed a unique material for the 3D culture of β-cells, which allowed the spontaneous assembly of β-cells into 3D spheroids by controlling the cell–matrix interaction, (HSPG-FGF2 interaction on an MBP-FGF2 immobilized surface) [39, 40]. Furthermore, this platform contributed to cell–matrix interaction, 3D structure formation, as well as stimulation of the FGF2 signaling cascade.

In this study, by harnessing FGF2-conjugated MBP with a hydrophobic region and HSPG binding affinity, we engineered a novel cell culture platform that controls the cell–matrix interaction-mediated promotion of β-cell differentiation and function. As previously reported, heparan sulfate (HS) is distributed and concentrated in the intercellular region within human islets of Langerhans [46]. Furthermore, HS is known to strongly affect β-cell proliferation and function as well as regulate the availability of growth factors at active sites [47, 48]. Moreover, FGF2 has a high affinity for FGF receptors (FGFR1–4) and HSPG, which form the FGF2-FGFR-HSPG complex [49]. Previously, we demonstrated that MBP-FGF2 interacts with cell surface HSPG and FGFR1, and that the FGF2-HSPG-FGFR1 complex initiates a signaling cascade [50]. FGFR1 signaling is important during pancreatic development, and previous studies have demonstrated that FGFR1 promotes PDX-1 expression during the early developmental stage of pancreatic progenitors, as demonstrated through inhibition experiments using an FGFR1 inhibitor and shFGFR1. When FGFR1 is inhibited, early-stage development and PDX-1 expression are also inhibited [45]. Similarly, we observed the cell-FGF2 interactions using HSPG-FGF2; when cells were cultured on the MBP-FGF2 surface, the SDC4 and TGF-βR3 gene expressions were increased compared with those in cells cultured on RBP during β-cell differentiation. We observed that when cells underwent differentiation on the MBP-FGF2 surface, PDX-1 expression was upregulated compared with that observed in cells cultured on RBP. These results suggest that cells bind to MBP-FGF2 through SDC4 and TGF-βR3 (an HSPG and a β-glycan, respectively) and that the HSPG-FGF2 complex upregulates PDX-1, resulting in the promotion of β-cell differentiation.

The pancreas is a multicellular organ consisting of β-cells that respond to elevated blood glucose levels by secreting insulin [23]. The gap junction protein connexin is related to cell–cell coupling, which contributes to the maturation and insulin secretion of pancreatic cells. In addition, a previous study showed that the gap junction-mediated electrical communication between β-cells in islets is critical for insulin secretion [51]. Together, these findings indicate that a high degree of cell–cell communication between β-cells promotes insulin secretion. Carvalho et al. investigated the localization of connexins such as Cx36 and the gap junction-mediated β-cell coupling in the pancreas and discovered that Cx36 was localized on β-cells with enhanced maturation and insulin secretion, which was explained by the intercellular exchange of cationic molecules between cells [25]. In addition, the role of Cx43 in the development and function of the human pancreas has been investigated, and previous reports have demonstrated that AAP10-induced promotion of Cx43 encourages the formation of Cx43 channels and improves the differentiation of embryonic stem cells into pancreatic cell lineages, indicating that Cx43-mediated signaling is important for the early development of pancreatic progenitors [52]. Therefore, in our study, we assessed cell–cell junction-associated markers, including Cx36 and Cx43, at both gene and protein levels. Our results corroborated the findings from previous studies as cells cultured on MBP-FGF2 exhibited significantly upregulated Cx36 and Cx43 gene and protein expression levels compared with those observed in RBP-cultured cells.

Cell aggregation-mediated formation of cell clusters is important for the secretion of insulin by β-cells [53, 54]. Additionally, a previous report showed that FGF2 promotes the aggregation of pancreatic precursor cells, rendering it essential for early cell cluster formation in precursor cells [55]. These results indicate that FGF2 is an important factor in stimulating the initial process of cell aggregation and clustering, leading to islet formation from pancreatic precursor cells, and ultimately underlying the important processes involved in the pancreatic differentiation and development. Cell adhesion molecules (CAMs) are also localized in pancreatic islet cells and play an important role in cell clustering [56]. Therefore, we assessed cell–cell adherent-junction-associated marker gene and protein expressions, including E-cadherin and Ncam1, using qPCR and IF, respectively. Ncam is a calcium-independent cell adhesion molecule expressed in islets, and Ncam expression is localized between islet cells during cell–cell interactions, which are necessary for cadherin clustering and islet development [57, 58]. E-cadherin is a type of CAM that has been found to be important in the formation of cell–cell adherent junctions, allowing cell–cell adherence, E-cadherin expression was also shown to be correlated with GSIS [59–61]. Furthermore, the expression of E-cadherin was increased in the pseudo-islets of MIN6 cells, and E-cadherin is necessary for islet cell organization [61–63]. As reported previously [64], our results showed that cells differentiated on the surface of MBP-FGF2 showed increased cell–cell interactions, which was confirmed by the upregulated gene and protein expression of E-cadherin and Ncam1. Additionally, the transcript-level expression level of Nkx6.1, Ucn3, and MAFA, known as β-cell maturation markers, was upregulated in MBP-FGF2-stimulated β-cells.

In a previous study, Hogrebe et al. developed a multi-stage-based novel protocol in two-dimensional culture to attain highly functional hPSC-derived β-cells by modulating actin polymerization using latrunculin A during the endocrine lineage commitment of hPSCs, instead of the conventional method that utilizes a bioreactor-based 3D suspension culture method [65]. More importantly, their in vivo results revealed that these functional β-cells derived from their novel protocol exhibited a significantly higher insulin SI and consequently reduced blood glucose levels more rapidly at an early stage, compared to those with β-cells differentiated using the conventional method. Further, previous studies on β-cells differentiation from stem cells have shown that both insulin-producing cells differentiated from stem cells and adult islets exhibit GSIS indices of 2–3 under low and high-glucose concentrations [8]. Similarly, in our study, cells differentiated on the MBP-FGF2 surface showed a higher glucose-responsive insulin SI compared to those from the RBP group. For examples, in our GSIS results, the MBP-FGF2 group showed an index close to 2.7, whereas the RBP group exhibited a relatively low index of 1.8. This observation suggests that the MBP-FGF2 group might have exhibited increased insulin secretion in response to glucose in mice with STZ-induced diabetes. This further indicates the possibility of faster blood glucose regulation at the initial timepoint in the MBP-FGF2 group. This may also have been achieved by the accelerated vascularization and neoangiogenesis around the transplanted β-cells. This is in agreement with previously reported studies that have highlighted the important role of vascularization and angiogenesis around β-cells in promoting insulin secretion and enhancing in vivo β-cell function [66, 67]. Furthermore, our previous study demonstrated that the formation of spheroids using human adipose-derived MSCs (hASCs) on the MBP-FGF2 cell culture platform upregulated in vivo engraftment by promoting the secretion of various angiogenic factors, including interleukin-8 (IL-8), fibroblast growth factors (FGFs), vascular endothelial growth factor (VEGF), and platelet-derived growth factors (PDGFs) [50, 68, 69]. When these FGF2-primed hASC spheroids were transplanted into an ischemic mouse model, cells could robustly secrete IL-8 through the FGFR1/JNK/NF-κB signaling cascade and contribute to therapeutic angiogenesis. Similarly, FGF2-immobilized matrix-assisted hASC spheroids have been shown to effectively reduce the size of myocardial infarction and inhibit apoptosis of cardiomyocytes by upregulating in vivo neoangiogenesis [70]. Taken together, the MBP-FGF2-immobilized cell culture platform may contribute to in vitro β-cell differentiation and maturation through cell–cell interactions, and consequently improve in vivo functional engraftment of stem cells by promoting angiogenesis, thus suggesting its potential biomedical application as a useful tool for the development of therapeutics and functional biomaterials.

## Conclusion

Our results demonstrated that the MBP-FGF2-based platform facilitated the formation of clusters that could differentiate into functional β-cells. This could be achieved through the upregulation of various cell–cell interacting proteins such as Cx36, Cx43, E-cadherin, and Ncam1, leading to enhanced in vitro differentiation of hO-MSCs into β-cells and GSIS. More importantly, our in vivo results clearly demonstrated that the hO-MSC-derived β-cells could effectively control hyperglycemia in mice with STZ-induced diabetes. Therefore, our findings suggest that the MBP-FGF2-based platform can promote β-cell differentiation and GSIS by supporting HSPG-FGF2-mediated cell adhesion and facilitating the formation of cell clusters through connexin and CAM-mediated cell–cell interactions among β-cells. This highlights the potential of the MBP-FGF2-based platform for promoting β-cell differentiation, maturation, and GSIS in therapeutic applications for T1DM. Such a cell culture platform can offer novel strategies to obtain functional pancreatic β-cells from patient-specific cell sources, ultimately enabling better treatments for diabetes mellitus.

### Supplementary Information

**Additional file 1:** **Supplementary Figure S1.** Differentiation potential of hO-MSCs into mesoderm lineages. (A) Adipogenic marker gene expression as well as Oil Red O and perilipin staining during the adipogenesis of hO-MSCs. (B) Osteogenic marker gene expression as well as Alizarin Red and osteocalcin (OCN) staining during the osteogenesis of hO-MSCs. (C) Chondrogenic marker gene expression as well as Alcian blue and type 2 collagen (COL II) staining during the chondrogenesis of hO-MSCs. Gene expression as well as cytochemical and immunofluorescence staining analyses were performed at the beginning of culture (day 0) and on the last differentiation day. Data represent the mean ± SD, *p < 0.05; **p < 0.01; ***p < 0.001. Scale bar = 200 μm. **Supplementary Figure S2.** Variations of body weight in each group across different time points. Ctrl (*n* = 5), STZ (*n* = 5), Sham (*n* = 7), RBP (*n* = 8), MBP-FGF2 (*n* = 8). Data represent the mean ± SEM, *p < 0.05; **p < 0.01; ***p < 0.001 comparing the mean of each group with the mean of the control (Ctrl: white, STZ: gray, Sham: green, RBP: red, MBP-FGF2: blue). #*p* < 0.05; ##*p* < 0.01; comparing the mean of the RBP group with the mean of the MBP-FGF2 group. RBP, round-bottom plate; MBP-FGF2, maltose-binding protein-basic fibroblast growth factor 2; STZ, streptozotocin.** Supplementary Figure S3.** Tissue gross images and histological analysis of the kidney. (A) Images of the kidney after 30 days (scale bar = 2 mm). (B) H&E staining and (C) immunofluorescence staining images of DAPI (blue), human-specific lamin A/C (LMNA) (red), and insulin (green). scale bar = 500 μm. RBP, round-bottom plate; MBP-FGF2, maltose-binding protein-basic fibroblast growth factor 2; STZ, streptozotocin. **Supplementary Table S1.** List of primers used for quantitative PCR.

## Data Availability

Please contact the corresponding author for raw data requests.
