# Supplementary material for: Self-organized insulin-producing β-cells differentiated from human omentum-derived stem cells and their in vivo therapeutic potential
Source: Biomater Res. 2023 Aug 29;27:82. doi: 10.1186/s40824-023-00419-1 (PMC10466773; doi:10.1186/s40824-023-00419-1)
Supplement: Supplementary file 1 — Additional file 1: Supplementary Figure S1. Differentiation potential of hO-MSCs into mesoderm lineages. (A) Adipogenic marker gene expression as well as Oil Red O and perilipin staining during the adipogenesis of hO-MSCs. (B) Osteogenic marker gene expression as well as Alizarin Red and osteocalcin (OCN) staining during the osteogenesis of hO-MSCs. (C) Chondrogenic marker gene expression as well as Alcian blue and type 2 collagen (COL II) staining during the chondrogenesis of hO-MSCs. Gene expression as well as cytochemical and immunofluorescence staining analyses were performed at the beginning of culture (day 0) and on the last differentiation day. Data represent the mean ± SD, *p < 0.05; **p < 0.01; ***p < 0.001. Scale bar = 200 μm. Supplementary Figure S2. Variations of body weight in each group across different time points. Ctrl (n = 5), STZ (n = 5), Sham (n = 7), RBP (n = 8), MBP-FGF2 (n = 8). Data represent the mean ± SEM, *p < 0.05; **p < 0.01; ***p < 0.001 comparing the mean of each group with the mean of the control (Ctrl: white, STZ: gray, Sham: green, RBP: red, MBP-FGF2: blue). #p < 0.05; ##p < 0.01; comparing the mean of the RBP group with the mean of the MBP-FGF2 group. RBP, round-bottom plate; MBP-FGF2, maltose-binding protein-basic fibroblast growth factor 2; STZ, streptozotocin. Supplementary Figure S3. Tissue gross images and histological analysis of the kidney. (A) Images of the kidney after 30 days (scale bar = 2 mm). (B) H&E staining and (C) immunofluorescence staining images of DAPI (blue), human-specific lamin A/C (LMNA) (red), and insulin (green). scale bar = 500 μm. RBP, round-bottom plate; MBP-FGF2, maltose-binding protein-basic fibroblast growth factor 2; STZ, streptozotocin. Supplementary Table S1. List of primers used for quantitative PCR. [file 40824_2023_419_MOESM1_ESM.docx]

**
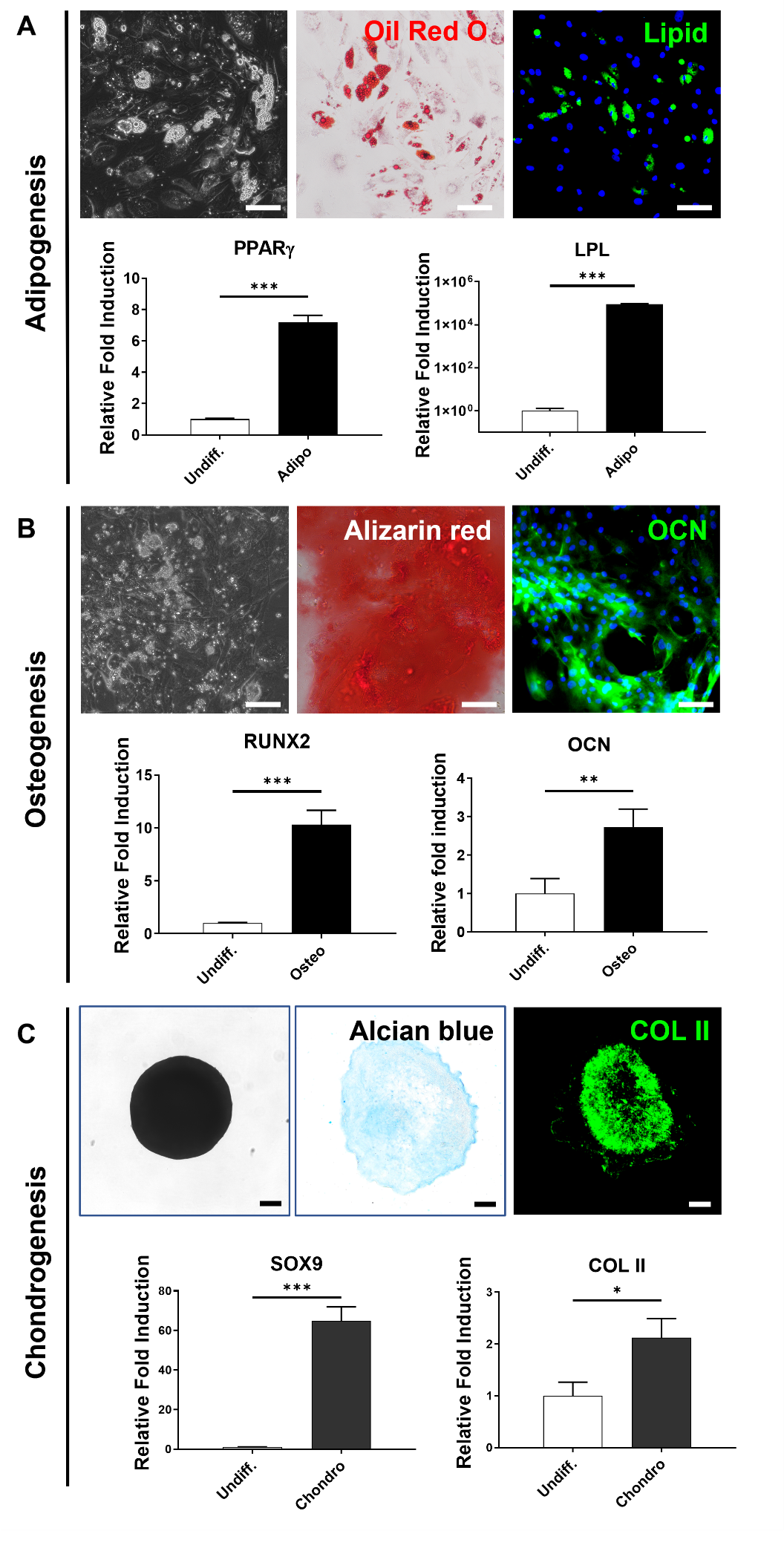
**

**Supplementary Figure S1.** Differentiation potential of hO-MSCs into mesoderm lineages. (A) Adipogenic marker gene expression as well as Oil Red O and perilipin staining during the adipogenesis of hO-MSCs. (B) Osteogenic marker gene expression as well as Alizarin Red and osteocalcin (OCN) staining during the osteogenesis of hO-MSCs. (C) Chondrogenic marker gene expression as well as Alcian blue and type 2 collagen (COL II) staining during the chondrogenesis of hO-MSCs. Gene expression as well as cytochemical and immunofluorescence staining analyses were performed at the beginning of culture (day 0) and on the last differentiation day. Data represent the mean ± SD, **p* < 0.05; ***p* < 0.01; ****p* < 0.001. Scale bar = 200 μm.


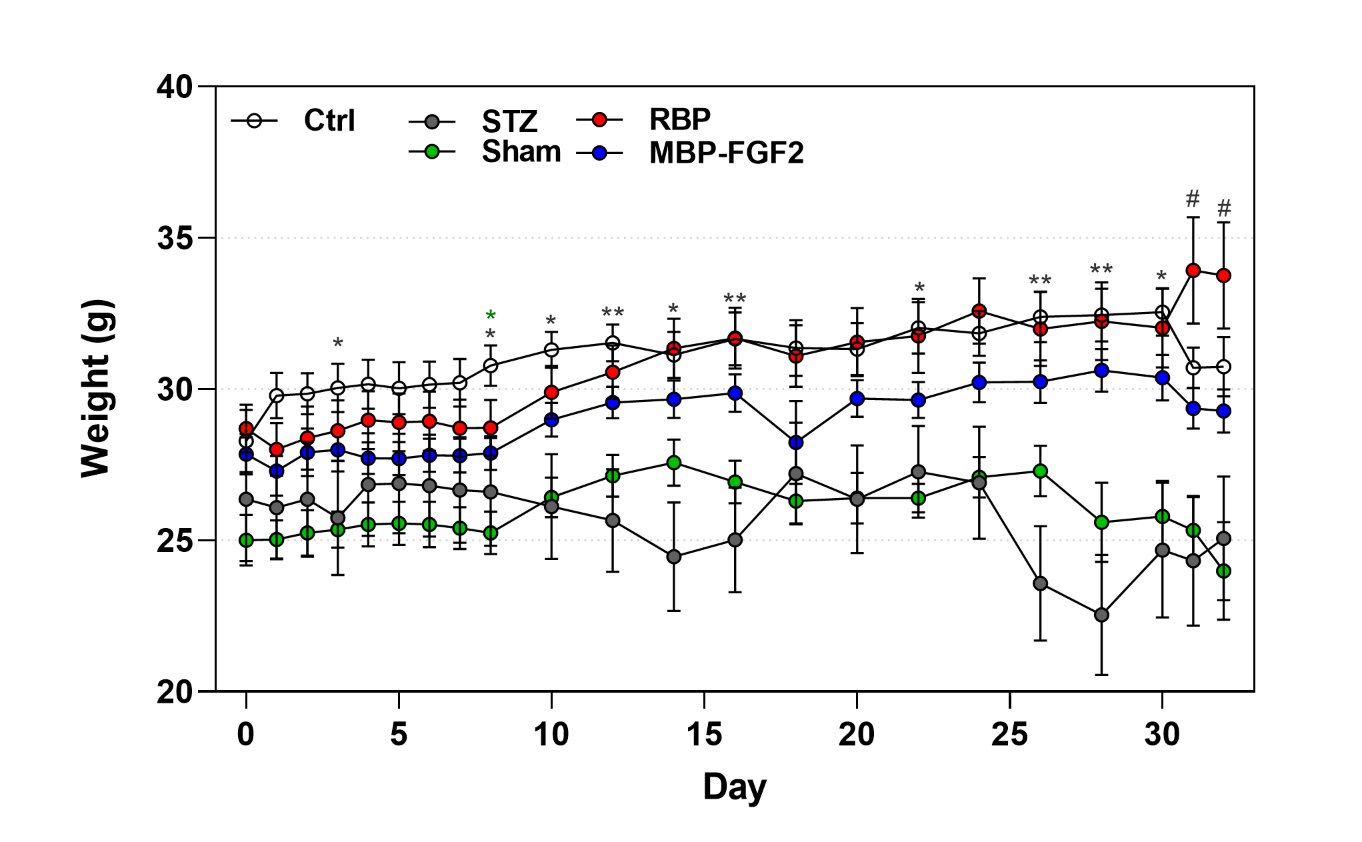


**Supplementary Figure S2.** Variations of body weight in each group across different time points. Ctrl (n = 5), STZ (n = 5), Sham (n = 7), RBP (n = 8), MBP-FGF2 (n = 8). Data represent the mean ± SEM, **p* < 0.05; ***p* < 0.01; ****p* < 0.001 comparing the mean of each group with the mean of the control (Ctrl: white, STZ: gray, Sham: green, RBP: red, MBP-FGF2: blue). #*p* < 0.05; ##*p* < 0.01; comparing the mean of the RBP group with the mean of the MBP-FGF2 group. RBP, round-bottom plate; MBP-FGF2, maltose-binding protein-basic fibroblast growth factor 2; STZ, streptozotocin.


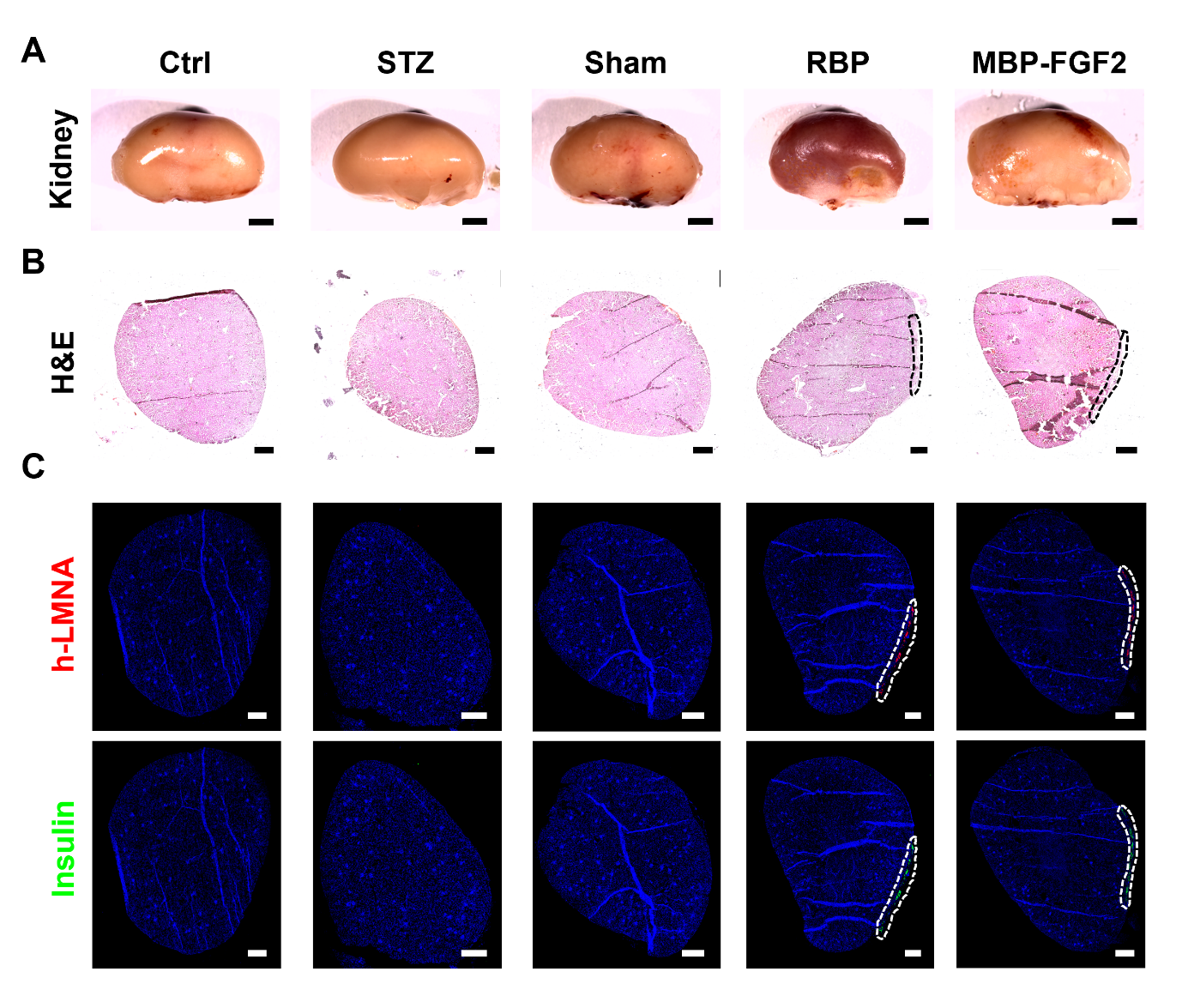
**Supplementary Figure S3. Tissue gross images and histological analysis of the kidney.** (A) Images of the kidney after 30 days (scale bar = 2 mm). (B) H&E staining and (C) immunofluorescence staining images of DAPI (blue), human-specific lamin A/C (LMNA) (red), and insulin (green). scale bar = 500 μm. RBP, round-bottom plate; MBP-FGF2, maltose-binding protein-basic fibroblast growth factor 2; STZ, streptozotocin.

| Gene | Primer Sequence (5′ to 3′) |
| --- | --- |
| *GAPDH* | F- CACTCCACCTTTGACGC |
|  | R- GGTCCAGGGGTCTTACTCC |
| *PAX4* | F- AGTCCTGCGGGCATTACAG |
|  | R- GGGAGAAGATAGTCCGATTCCG |
| *NGN3* | F- GCTGCTCATCGCTCTCTATTCTT |
|  | R- CGAGGGTTGAGGCGTCAT |
| *NKX2.2* | F- GGAGCGCCACGAATTGAC |
|  | R- TTCGAGACCCCAAAATTTATGTC |
| *PDX1* | F- TTTCTATTTAGGATGTGGACGTAATTCC |
|  | R- GGCCACTGTGCTTGTCTTCA |
| *INS1* | F- GCAGCCTTTGTGAACCAACA |
|  | R- TTCCCCGCACACTAGGTAGAGA |
| *GLUT2* | F- TTTTTCAGACGGCTGGTATCAG |
|  | R- CCATGTTTACAGCGCCAACTC |
| *UCN3* | F- CCCACAAGTTCTACAAAGCCA |
|  | R- TCCCGAAGAGGCGTCTCTG |
| *NKX6.1* | F- ACCCCTCATCAAGGATCCATT |
|  | R- TGGGTCTCGTGTGTTTTCTCTTC |
| *MAFA* | F- GAGCGGCTACCAGCATCAC |
|  | R- CTCTGGAGTTGGCACTTCTCG |
| *CX36* | F- GGGGCAAACGAGAAGATAAGAA |
|  | R- TGGATAATGTAGAAGCGGGAGA |
| *CX43* | F- TGGTAAGGTGAAAATGCGAGG |
|  | R- GCACTCAAGCTGAATCCATAGAT |
| *NCAM1* | F- GGCATTTACAAGTGTGTGGTTAC |
|  | R- TTGGCGCATTCTTGAACATGA |
| *E-CAD* | F- AGGTGACAGAGCCTCTGGATAGA |
|  | R- GGATGACACAGCGTGAGAGAAG |
| *SDC4* | F- GGACCTCCTAGAAGGCCGATA |
|  | R- TCCAGAGCCAGACAGCTCAA |
| *TGF-βR3* | F- TGGGGTCTCCAGACTGTTTTT |
|  | R- CTGCTCCATACTCTTTTCGGG |
| *PPARγ* | F- ACCAAAGTGCAATCAAAGTGGA |
|  | R- ATGAGGGAGTTGGAAGGCTCT |
| *LPL* | F- AGGATGTGGCCCGGTTTATC |
|  | R- CCAAGGCTGTATCCCAAGAGAT |
| *RUNX2* | F- CCACCCGGCCGAACTGGTCC |
|  | R- CCTCGTCCGCTCCGGCCCACA |
| *OCN* | F- TGAGAGCCCTCACACTCCTC |
|  | R- CCTCCTGCTTGGACACAAAG |
| *SOX9* | F- AGCGAACGCACATCAAGAC |
|  | R- CTGTAGGCGATCTGTTGGGG |
| *COLII* | F- GGCAATAGCAGGTTCACGTACA |
|  | R- CGATAACAGTCTTGCCCCACTT |

**Supplementary Table S1.** List of primers used for quantitative PCR
